# Prevalence Rate of Hepatitis C Among the Solid Waste Handler in Wardha City

**DOI:** 10.7759/cureus.19888

**Published:** 2021-11-25

**Authors:** Mayur B Wanjari, Deeplata Mendhe

**Affiliations:** 1 Community Health Nursing, Smt. Radhikabai Meghe Memorial College of Nursing, Wardha, IND; 2 Community Health Nursing, Datta Meghe Institute of Medical Sciences, Wardha, IND

**Keywords:** cross- sectional research design, solid waste handlers, prevalence, hepatitis c, hiv

## Abstract

Introduction:

Solid waste workers are exposed to an extended variety of occupational hazards. Among these hazards is the infection from hepatitis A, B, or C viruses (HAV, HBV, or HCV). This relationship has been the study subject of many researchers around the world, given that the infection of hepatitis viruses is a significant cause of morbidity and a socio-economic burden. Solid waste handlers are usually at significant risk for multiple injuries and illnesses, including HIV and hepatitis, due to waste exposure to contaminated needles or sharp items that may contribute to the spread of the disease. A research in Brazil revealed that 12.8% of HBV exposure is prevalent in municipal solid waste handlers.

Objectives:

To assess the prevalence rate of hepatitis C among the solid waste handler in selected areas and associate the findings with selected demographic variables.

Material and methods:

This study was used as a cross-sectional research design. Hundred solid waste handlers participated in the study. The prevalence of hepatitis C was checked by the blood sampling and use method: HCV Ab Rapid Test. Data were analyzed using the IBM Corp. Released 2016. IBM SPSS Statistics for Windows, Version 24.0. Armonk, NY: IBM Corp. Qualitative variables were described as numbers and percentages. Chi-square exact test was used for comparison between groups, as a quantitative variable was described as mean (± SD) and median.

Results:

10 (10%) of the waste handlers were reactive to hepatitis C virus, and 90 (90%) of the waste handlers were non-reactive to hepatitis C virus. The mean was 1.92 ± 0.27 for the prevalence of hepatitis C among solid waste handlers.

Conclusion:

A high prevalence of hepatitis C is revealed, particularly in people who have more working experience, exposure, and who do not use personal protective equipment while working around hepatitis C infected people. It is recommended that all the solid waste handlers use proper personal protective equipment, go for routine health check-ups, and should be trained on handling waste to reduce morbidity.

## Introduction

An unavoidable result of the production and treatment of waste material is the aerosolization of microbial bacteria, endotoxins, odors, and dust particles. Primary human pathogens (including viruses, mycoplasmas, microbes, fungi, and intestinal parasite cysts or eggs, primarily contained in disposable diapers and household medical waste), secondary pathogens and their toxins (spores and endotoxins) created by domestic (composting) waste bacteria and fungi formation, volatile and semi-volatile organic chemicals, both synthetic and natural origin, household and yard waste allergens, corrosive, caustic, explosive and sharp materials are household waste that is present. Any of the most widely recorded occupational health and injury issues for MSW employees are respiratory diseases (due to particulate, bioaerosol, and volatile organic inhalation during waste collection), infections (due to direct contact with infected products, dog and mouse bites, or feeding waste fed animals), perforated wounds (may contribute to HIV, tetanus, and hepatitis), headache and nausea (due to noxious content of waste and odor). Physically exhausting work carried out at high-speed results in a pulmonary ventilation rate of 25-40 l/min instead of the usual 6 l/min. Thirty nine particles can move farther down the respiratory tract at high pulmonary ventilation, thus causing an irritative reaction [1].

According to the International Labor Organization, of 2002, 270 million workers face accidents in the workplace every year (approximately 360 000 fatal), and another 160 million workers suffer from occupational illnesses, with millions dying every year (ILO-2002). While the risk of staff is generally higher than in the general public by exposure, the dangers and threats are usually not directly investigated during the health impact assessment. For other aspects of waste disposal, the storage of municipal solid waste constitutes the largest and most complex group of hazards. There are insufficient research and limited data on solid waste workers and the resulting health impacts [2].

Also, there are reports from different countries suggesting that injuries caused by sharp objects were much higher among municipal waste collectors from the USA, Brazil, and Taiwan than among their Danish colleagues. Hepatitis B Virus (HBV) is resistant to environmental surfaces for at least seven days. Consequently, municipal collectors could have a theoretical risk of acquiring HBV infection after exposure to contaminated sharp instruments that have been improperly discarded. This could also be the case for hepatitis C virus infection (HCV). Up today, there are only two studies that reported a positive association between exposures to sharp instruments during the waste collection process and the risk of HBV infection. Additionally, there is a scarcity of data on the prevalence and risk factors for HCV infection among municipal waste workers [3].

## Materials and methods

Study setting and design

The cross-sectional study was conducted at Ram Nagar and Krishna Nagar at Wardha city between July to Aug 2021. Solid waste handlers of these areas were selected for the research study.

Study population

Purposive sampling was taken into this study. A total of 100 solid waste handlers were selected in this research. Inclusion criteria for the study are those solid waste handlers present at the time of the research and willing to participate in this research. 

Data collection

The most crucial aspect of any investigation is collecting adequate information as data that will help to answer the question raised in the study, which is the prevalence of hepatitis C among solid waste handlers. Blood samples were taken for a detailed investigation. A good rapport was maintained. On the first day, each participant was given a questionnaire and asked to fill in some demographic variable information like age in years, gender, educational status, work experience, source of collection waste, personal protective equipment, etc. The questionnaire was collected soon after filling it up. On the same day, the hepatitis C estimation was done by HCV Ab Rapid Test.

Statistical analysis

Data collected were analyzed using Statistical Package for Social Sciences (IBM Corp. Released 2016. IBM SPSS Statistics for Windows, Version 24.0. Armonk, NY: IBM Corp.) Demographic data were collected using a questionnaire, which was developed by the researchers for the study. The mean score was calculated and rated. Chi-square was used to determine the association between dependent (hepatitis C) and independent variables (socio-demographic profile). The level of statistical significance was achieved if p<0.05 at a 95% confidence level.

Sample collection and laboratory analysis

Two milliliters (2mls) of blood collected was aseptically obtained from study participation by venepuncture and dispensed into the bare bulb. A trained phlebotomist carried out blood sample collection. Samples were placed in an airtight container and transported in sealed bags. After the samples reached the laboratory HCV Ab Rapid Test was conducted. 

Ethical consideration

Written informed consent was obtained from each participant after a careful explanation of the concept and purpose of the study. The participants were ensured of privacy and confidentiality. The study protocol was reviewed and approved by the DMIMS(DU)/IEC/Dec-2019/8672.

## Results

Demographic characteristics of the study population

This section deals with the percentage-wise distribution of solid waste handlers according to their demographic characteristics. The data obtained to describe the sample characteristics include age, gender, educational status, working experience in a year, and source of waste collection (Table 1).

**Table 1:** Percentage-wise distribution of solid waste handlers according to their demographic characteristics.

Demographic Variable	Frequency	Percentage
Age in year		
18-28	27	27%
29-39	26	26%
40-50	29	29%
51 above	18	18%
Gender		
Male	53	53%
Female	47	47%
Educational status		
Primary education	18	18%
Secondary education	51	51%
Higher secondary education	26	26%
Graduate	05	05%
Postgraduate	0	0%
Working experience in year		
0-5	14	14%
6-10	27	27%
11-15	15	15%
16-20	17	17%
21-25	10	10%
26-30	06	06%
31-35	07	07%
36-40	04	04%
Sources of collected waste		
Residential	54	54%
Commercial	25	25%
Industrial	09	09%
Institutional	12	12%
Do you use personal protective equipment?		
Yes	40	40%
No	60	60%
Do you have a history of blood transfusion in the last 4-10 Weeks?		
Yes	02	02%
No	98	98%
Do you already diagnose with HCV?		
Yes	02	02%
No	98	98%

Hepatitis C status of the study population

Mean was 1.92 ± 0.27 of the prevalence of hepatitis C among solid waste handlers, 10 (10%) of the waste handlers were reactive to hepatitis C virus and 90 (90%) of the waste handlers were non-reactive to hepatitis C virus (Table 2).

**Table 2:** Prevalence of Hepatitis C among solid waste handlers.

Score Range	Prevalence of Hepatitis C		
	Frequency	Percentage	Mean ± SD
Reactive	10	10%	1.92 ± 0.27
Non-reactive	90	90%	

Associate the findings with selected demographical variables

There were significant differences in the distributions of working experience, personal protective measures, and being already diagnosed with HCV (p = 0.030; p = 0.004; p= 0.009, respectively). Whereas there was no significant difference in age, gender, educational status, source of waste collection, and history of blood transfusion in the last 4-10 weeks (Table 3).

**Table 3:** Associate the findings with selected demographical variables

Demographic Variable	Frequency	HCV		א2-value	df	א2-tab value	p-value
Age in year		Positive	Negative				
18-28	27	02	25	3.503	3	5.164	0.320 NS
29-39	26	04	22				
40-50	29	04	25				
51 above	18	0	18				
Gender	Frequency	Positive	Negative				
Male	53	06	47	0.219	1	0.018	0.449NS
Female	47	04	43				
Educational status	Frequency	Positive	Negative				
Primary education	18	01	17	7.153	3	9.761	0.067 NS
Secondary education	51	09	42				
Higher secondary education	26	0	26				
Graduate	05	0	05				
Postgraduate	0	0	0				
Working experience in year	Frequency	Positive	Negative				
0-5	14	01	13	15.475	7	13.333	0.030 S
6-10	27	01	26				
11-15	15	01	14				
16-20	17	06	11				
21-25	10	01	09				
26-30	6	0	06				
31-35	7	0	07				
36-40	4	0	04				
Sources of collected waste	Frequency	Positive	Negative				
Residential	54	09	45	6.790	3	10.077	0.079 NS
Commercial	25	0	25				
Industrial	9	01	08				
Institutional	12	0	12				
Do you use personal protective equipment?	Frequency	Positive	Negative				
Yes	40	0	40	7.407	1	5.671	0.004 S
No	60	10	50				
Do you have a history of blood transfusion in the last 4-10 weeks?	Frequency	Positive	Negative				
Yes	2	0	02	0.227	1	0.001	0.809 NS
No	98	10	88				
Do you already diagnose with HCV?	Frequency	Positive	Negative				
Yes	2	02	0	18.367	1	9.580	0.009 S
No	98	08	90				

## Discussion

In the present study, hepatitis C is identified when the solid waste handlers are exposed to the hepatitis C virus. The analysis found that 10 (10%) of solid waste handlers had a reaction to the hepatitis C virus and 90 (90%) of solid waste handlers were non-reactive to hepatitis C virus.

The study is supported by a descriptive cross-sectional survey of municipal solid waste (MSW) collectors (n=120) working in the Western Municipality of Mansoura, Egypt. An MSW collector was found with a high prevalence (43.3%) of HCV antibodies. Statistically significant demographic variables of seropositivity of HCV antibodies were the older age and longer duration of collector employment. The logistic regression analysis showed that a lower probability of HCV antibody seropositivity (OR=0.3) was independently associated with the shorter duration of work as a waste collector [4].

Santos, south-eastern Brazil support the study; Rozman et al. found that the seroprevalence of HCV infection among recyclable waste collectors was 12.4%. The majority of the study participants were males with low educational and economic levels, exposed to HIV and other sexually transmitted infections through parenteral and sexual exposure, and 10% of them had used intravenous drugs. This occurrence is estimated to be 10 to 12 times higher than the national average, according to the findings [5].

The study is supported by a recent study by Rachiotis et al. of MSW workers in a municipality of central Greece who reported that the prevalence of HBV infection was 23%. The logistic regression analysis showed that the anti-HBc positivity was independently associated with exposure to waste (OR =4.05; 95 % CI=1.23-13.33) and age (OR=5.22; 95 % CI= 1.35-20.1). Also, the risk of HBV infection was higher among waste collectors with needle sticks who reported occupational injuries (RR=2.64; 95 % CI= 1.01-6.96). The independent predictors recorded in the analysis are close to our study results [6].

In India, hepatitis C is an emerging infection that will have long-term effects in the coming decades. In different regions of India, it is a pathogen that is already responsible for a large portion of liver disease. The advent of the HIV epidemic may add to the existing burden of HCV infection in the country. It is important to enforce strict blood banking laws and prohibit the sterilization and reuse of needles. All of this is inevitable without a more extensive public understanding of the scope and effects of this chronic infection, as well as its mode of transmission. Hepatitis C must be on the radar of health officials as a disease that has the potential to cause substantial morbidity and mortality in the future [7].

Due to their exposure to sharp injuries without any safety measures, solid waste collectors are at a higher risk of HCV infection than the general population. Solid waste collectors are economically oppressed and typically not regarded by national initiatives as potentially endangered populations. There is a need for further research through interventions to reduce the high prevalence of Anti-HCV among collectors.

Limitation of the study

The limitation of these studies is the small sample size and reliability of the confirmation by the rapid test estimation.

## Conclusions

Association of hepatitis C with demographic variables such as working experience near infected patients and absence of personal protective equipment along with other demographic variables are established. The end of our research project reveals the high prevalence of hepatitis C, particularly in those who have more working experience, exposure, and are not using personal protective equipment while working around hepatitis C infected people. It is recommended that all the solid waste handlers use proper personal protective equipment, go for routine health check-ups, and should be trained on handling waste to reduce morbidity.
